# Regulatory Role of IL-1R8 in Immunity and Disease

**DOI:** 10.3389/fimmu.2016.00149

**Published:** 2016-04-20

**Authors:** Martina Molgora, Isabella Barajon, Alberto Mantovani, Cecilia Garlanda

**Affiliations:** ^1^Department of Inflammation and Immunology, Humanitas Clinical and Research Center, Rozzano, Italy; ^2^Humanitas University, Rozzano, Italy

**Keywords:** cytokine, interleukin-1, toll-like receptors, inflammation, infection, inflammation-associated cancer

## Abstract

Interleukin-1 receptor family members (ILRs) and toll-like receptors (TLRs) are characterized by the presence of a conserved intracellular domain and the toll-IL-1resistance (TIR) domain and are key players in immunity and inflammation. ILR and TLR signaling is tightly regulated at different levels. All cell types of the innate immune system express ILRs and TLRs. In addition, IL-1 family members are emerging as key players in the differentiation and function of innate and adaptive lymphoid cells. IL-1R8, also known as TIR8 or SIGIRR, is a fringe member of the ILR family and acts as a negative regulator of ILR and TLR signaling, which dampens ILR- and TLR-mediated cell activation. IL-1R8 is a component of the receptor recognizing human IL-37. Here, we summarize our current understanding of the structure and function of IL-1R8, focusing on its role in different pathological conditions, ranging from infectious and sterile inflammation to autoimmunity and cancer-related inflammation.

## Introduction

**Figure 1:**
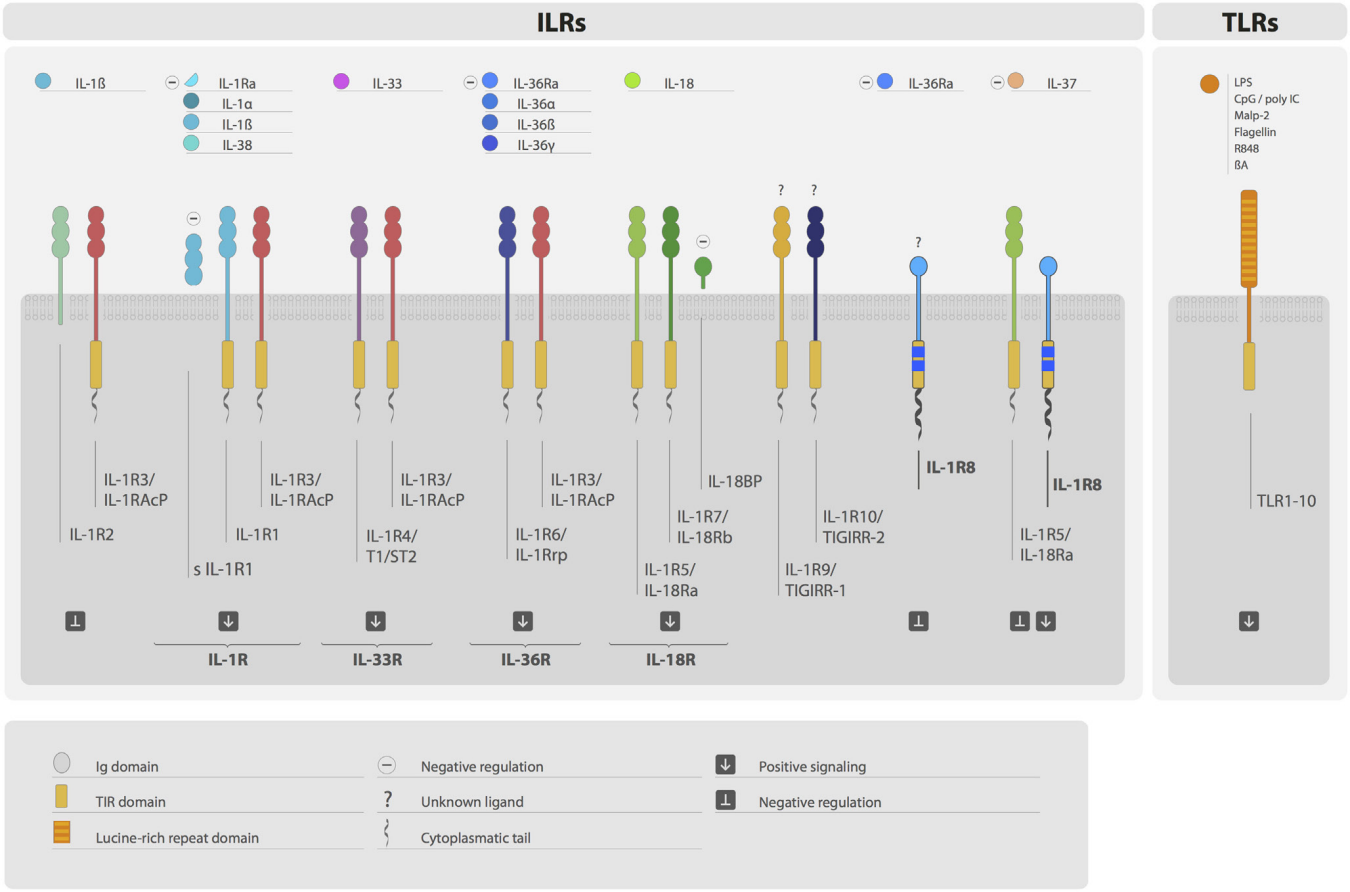
**The IL-1 receptor (ILRs) and toll-like receptor (TLRs) superfamily**. Ligands, receptors, accessory proteins, and regulators are shown. Ligands of the ILR family include IL-1α, IL-1β, IL-38, IL-33, IL-36α, IL-36β, IL-36γ, and IL-18. Microbial compounds (LPS, CpG, poly IC, flagellin, and others), β-amyloid, and danger signals are ligands for TLRs. IL-1R, IL-33R, IL-36R, and IL-18R complexes transduce positive signals. IL-R2, sIL-1R1, IL-1Ra, IL-36Ra IL-18BP, and IL-1R8 are negative regulators acting at different levels. IL-37 is an anti-inflammatory cytokine, which signal is dependent on the formation of a tripartite complex (IL-37/IL-1R5/IL-1R8). IL-1R3 is an accessory protein, which activity is necessary for IL-R1, IL-1R2, IL-1R4, and IL-1R6 function. IL-1R8, IL-1R9, and IL-1R10 are still orphan receptors.

Interleukin-1 receptor family members (ILRs) and toll-like receptors (TLRs) are members of a superfamily characterized by the presence of a common intracellular signaling domain, called toll-IL-1 resistance (TIR) domain, and Ig-like domains or leucine-rich repeats in their extracellular part (1) (Figure 1). ILRs and TLRs are phylogenetically conserved receptors responsible for the initiation and amplification of events leading to inflammation and innate and adaptive immune responses. TLRs work as sensors for exogenous infectious agents and host tissue injury, recognizing specific pathogen-associated molecular patterns (PAMPs) and damage-associated molecular patterns (DAMPs). Ten TLRs have been identified to date in humans and 12 in the mouse. The ILR subfamily is a growing family of receptors and accessory proteins (AcP) for the cytokines of the IL-1 family. The nomenclature of ILRs has recently been revised (2) and will be used here: IL-1R1 (IL-1RI), IL-1R2 (IL-1RII), IL-1R3 (IL-1RAcP), IL-1R4 (ST2), IL-1R5 (IL-18Rα), IL-1R6 (IL-1Rrp2, IL-36R), IL-1R7 (IL-18Rβ), IL-1R8 (also known as TIR8 or SIGIRR), IL-1R9 (TIGIRR-2), and IL-1R10 (TIGIRR-1). The IL-1 system includes molecules with agonist activity (IL-1α, IL-1β, IL-18, IL-33, IL-36α, IL-36β, and IL-36γ), three receptor antagonists (IL-1Ra, IL-36Ra, and IL-38), and an anti-inflammatory cytokine (IL-37) (Figure 1).

Upon ligand binding, TLRs and ILRs dimerize through their TIR domains, inducing the recruitment of TIR domain-containing adapter proteins, in particular MyD88, MAL, TRIF, TRAM, and SARM, which couple to downstream protein kinases [e.g., IL-1R-associated kinases (IRAKs) and tumor necrosis factor receptor-associated factor 6 (TRAF6)]. The signal leads to the activation of key transcription factors associated with inflammatory and immune responses, such as nuclear factor-κB (NFκB), activator protein-1 (AP-1), c-Jun N-terminal kinase (JNK), p38 mitogen-associated protein kinase, extracellular signal-regulated kinases (ERKs), mitogen-activated protein kinases (MAPKs), and members of the interferon (IFN)-regulatory factor (IRF) (3–5).

IL-1 family and TLR signaling are a crucial network that covers a wide spectrum of functions in several tissues and cell types, ranging from tissue homeostasis regulation to protective responses against infections and modulation of inflammation. Given the huge capacity of ILR and TLR triggering to drive inflammatory responses, the strict regulation of this system plays a significant role in both physiological and pathological conditions.

Both ILR and TLR functional activations are modulated by several and diverse mechanisms. Among these, IL-1R2 exerts regulatory functions acting as decoy receptor for IL-1, dominant-negative molecule, and scavenger (6, 7). In addition, it is also present in the cytoplasm where it binds pro-IL-1α and pro-IL-1β, preventing their cleavage and activation (8). IL-1R8, also known as TIR8 or SIGIRR, whose function will be detailed below, is a fringe member of the family that lacks conventional signaling capacities and behaves as a negative regulator of the family, acting intracellularly. Available information suggests that IL-1R8 interferes with the association of TIR-containing adaptor molecules to the receptor complex, thus dampening the signaling pathway leading to signal transduction (9, 10). In addition, IL-1R8 is a component of the receptor recognizing the anti-inflammatory cytokine IL-37 (11). IL-37 is an anti-inflammatory cytokine that acts as a natural brake of inflammation, signaling through IL-1R5/IL-18Rα and IL-1R8 was recently described as a coreceptor, required for the formation of the tripartite complex IL-37–IL-1R5/IL-18Rα–IL-1R8 (11). IL-18BP is an extracellular protein that binds IL-18, preventing its interaction with the receptor IL-1R5/IL-18R, and thus neutralizing its activity (12–14). IL-1Ra and IL-36Ra are receptor antagonists that bind IL-1R1 and IL-1R6, respectively (5, 15–18).

IRAK-M and MyD88s are intracellular signaling molecules that negatively regulate ILR and TLR pathways (19, 20). Finally, specific miRNAs (miR-155, miR-21, miR-146a, miR-132, miR-9, and miR-147) target ILR and TLR signaling proteins (21–23). The abundance of these regulatory mechanisms underlines the relevance of the negative regulation of both ILRs and TLRs, which if uncontrolled, may activate detrimental inflammation and cause tissue damage. For instance, local and systemic inflammation induced by IL-1 underlay a broad list of diseases, ranging from rheumatic diseases and autoinflammatory syndromes to cardiovascular diseases, type 2 diabetes, and infections and sepsis, and targeting of IL-1 has relevant therapeutic implications (24–27).

Here, we will review our current understanding of the structure and function of the negative regulator IL-1R8 (TIR8/SIGIRR), focusing on its relevance in different inflammatory or immune-mediated pathological disorders and emphasizing recent discoveries.

## IL-1R8 (TIR8/SIGIRR) Gene and Protein

IL-1R8 was identified by our group and reported as TIR8 in 1998 (Accession number: AF113795), and in parallel by John Sims’ group in 1999 and reported as SIGIRR (28). IL-1R8 is localized on human chromosome 11, band p15.5, and is composed of 10 exons spanning about 11,700 bp (28). It is therefore not part of the IL-1R family cluster, which is located in humans on chromosome 2. The murine gene is localized on chromosome 7, band F4, and encompasses nine exons spanning about 4000 bp. The human protein is 410 amino acids long and displays unique features compared with other ILRs. It is composed of a single extracellular Ig domain, in contrast with the other family members which have three, a transmembrane domain, a cytoplasmic TIR domain, and an unusually long tail (95 residues), which is missing in other TIR domain-containing receptors. The IL-1R8 TIR domain lacks two conserved residues (Ser447 and Tyr536), which are replaced by Cys222 and Leu305 suggesting unconventional signaling. IL-1R6 and IL-1R3 display a similar amino acid substitution in their TIR domain, but the functional relevance of this replacement has not been addressed yet (28, 29).

IL-1R8 sequence is highly conserved among vertebrates, from chicken to human in terms of sequence and pattern of expression (30). Human and murine protein sequences are 82% identical and share 23% overall identity with IL-1R1. In teleost fish, the receptor DIGIRR, which has two Ig-like domains in its extracellular region and an Arg–Tyr-mutated TIR domain, exerts regulatory activities *in vitro*, negatively modulating LPS, and IL-1β-dependent NFκB activation, therefore resembling a “transitional” form between the signaling molecule IL-1R1 and the negative regulator IL-1R8 (31).

IL-1R8 has five potential N-glycosylation sites in the extracellular domain in humans and four in the mouse and is extensively *N*- and *O*-glycosylated. Zhao et al. recently showed that loss of N-linked glycosylation was associated with an inactive isoform of IL-1R8, generated by alternative splicing in colon cancer cells. Moreover, loss of complex glycan modifications was sufficient to suppress IL-1R8 activity *in vivo*, highlighting that posttranscriptional modifications are required for the functional activity of IL-1R8 (32).

IL-1R8 is widely expressed in several epithelial tissues, in particular by epithelial cells of the kidney, digestive tract, liver, lung, and in lymphoid organs. Among leukocytes, it is expressed by monocytes, B and T lymphocytes, dendritic cells, and NK cells (28, 33). Little is known about the regulation of IL-1R8 expression and the stimuli and pathways involved. In general, both IL-1R8 mRNA and protein expression are reduced in inflammatory conditions. IL-1R8 was downmodulated in ulcerative colitis in humans and colitis in the mouse, intestinal bacterial infections, and exposure to flagellin (34, 35). The expression of IL-1R8 (and other anti-inflammatory molecules) was reduced in leukocytes of psoriatic arthritis patients and, together with TLR4, it was reduced in asymptomatic bacteriuria patients (36, 37). Nanthakumar et al. showed that IL-1R8 level was decreased in intestinal cells of necrotizing enterocolitis patients compared with fetal human enterocytes, whereas pro-inflammatory proteins were expressed at high levels, in line with the exacerbated inflammatory response of the immature intestine (38). In the mouse, acute lung infection by *Pseudomonas aeruginosa* caused *IL-1R8* mRNA downregulation in the lung and in neutrophils (39). IL-1R8 transcript was also reduced upon intestinal infection by *Toxoplasma gondii* (40). In a model of pyelonephritis induced by *Escherichia coli*, it was shown that renal IL-1R8 mRNA was downregulated in the early phase of infection and it started to return to basal level 24 h postinfection (41). Moreover, *in vitro* experiments in human bladder epithelial cells (BECs) demonstrated that IL-1R8 mRNA and protein expression were downregulated upon stimulation with LPS (42). Finally, colon tumorigenesis was shown to be associated with a lower expression level of IL-1R8 on the intestinal cell surface compared with the healthy counterpart. This was due to a mechanism of alternative splicing that caused the generation of an inactive mutant form of IL-1R8 that will be further discussed below (32).

Concerning the mechanism of IL-1R8 downregulation, Kadota et al. showed that LPS treatment reduced the binding between SP1, a zinc finger protein, and the proximal promoter of IL-1R8 (34). SP1 would normally directly interact with IL-1R8 promoter and favor gene transcription, but in presence of LPS the binding was inhibited and IL-1R8 expression transiently decreased in epithelial cells. Recently, the role of SP1 in the regulation of IL-1R8 mRNA expression was also confirmed in human primary monocytes and neutrophils. The inhibition of SP1 binding to IL-1R8 promoter induced by LPS was due to the activation of p38, which is downstream of TLR4. Indeed, treatment of both monocytes and neutrophils with a p38 inhibitor (SB203580) abolished the LPS-induced downregulation of IL-1R8 mRNA (43). Conversely, sepsis and sterile systemic inflammation have been associated with higher level of IL-1R8 expression by monocytes compared with homeostatic conditions, which correlated with reduced TNFα and enhanced IL-10 production in response to LPS and Pam_3_CysSK_4_ (44).

IL-1R8 is differentially expressed in polarized T lymphocytes. Murine Th2 cells displayed higher levels of IL-1R8 compared with Th1 or naive T cells (45). In *P. aeruginosa*-infected mice, IL-1R8 was upregulated by the neuropeptide vasoactive intestinal peptide (VIP) in a cAMP-independent manner in the cornea, in macrophages, and in Langerhans cells (46). The probiotic microorganism *Lactobacillus jensenii* was found to upregulate IL-1R8, *via* TLR2 in porcine Payer’s patch antigen-presenting cells, and to favor the expression of IL-10 and TGF-β, thus inducing tolerogenic properties (47). Finally, in murine Payer’s patch DCs, but not splenic DCs, LPS was shown to induce the upregulation of IL-1R8, Tollip, and IL-1R4. This could be a potential mechanism used by Payer’s patch DCs to modulate the inflammation driven by TLR signaling (48).

Recently, Costello et al. proposed a mechanism involved in IL-1R8 regulation in the context of neuroinflammation. Amyloid β treatment increased the expression of TLR2 and decreased the expression of IL-1R8 in microglia. Interestingly, TLR2 neutralization led to an increase of IL-1R8 mRNA in microglia and hippocampal tissue (49). The transcription factor peroxisome proliferator-activated receptor (PPAR)γ is a key anti-inflammatory mediator that regulates Aβ responses in the brain. Binding sites for the transcription factor PPARγ were identified in the IL-1R8 gene and treatment with the PPARγ inhibitor (GW9662) reduced the anti-TLR2-mediated IL-1R8 upregulation, supporting the involvement of PPARγ in the modulation of IL-1R8 expression. The PI3K/Akt pathway was also involved in the regulation of IL-1R8, since PI3K inhibitor (LY294002) abolished the effect of TLR2 neutralization. The expression of IL-1R8 and TLR2 is therefore inversely correlated and IL-1R8 upregulation mediated by TLR2 neutralization may be a compensation mechanism adopted to limit the deleterious effect of Aβ (49).

## IL-1R8-Mediated Anti-Inflammatory Activity of IL-37

**Figure 2:**
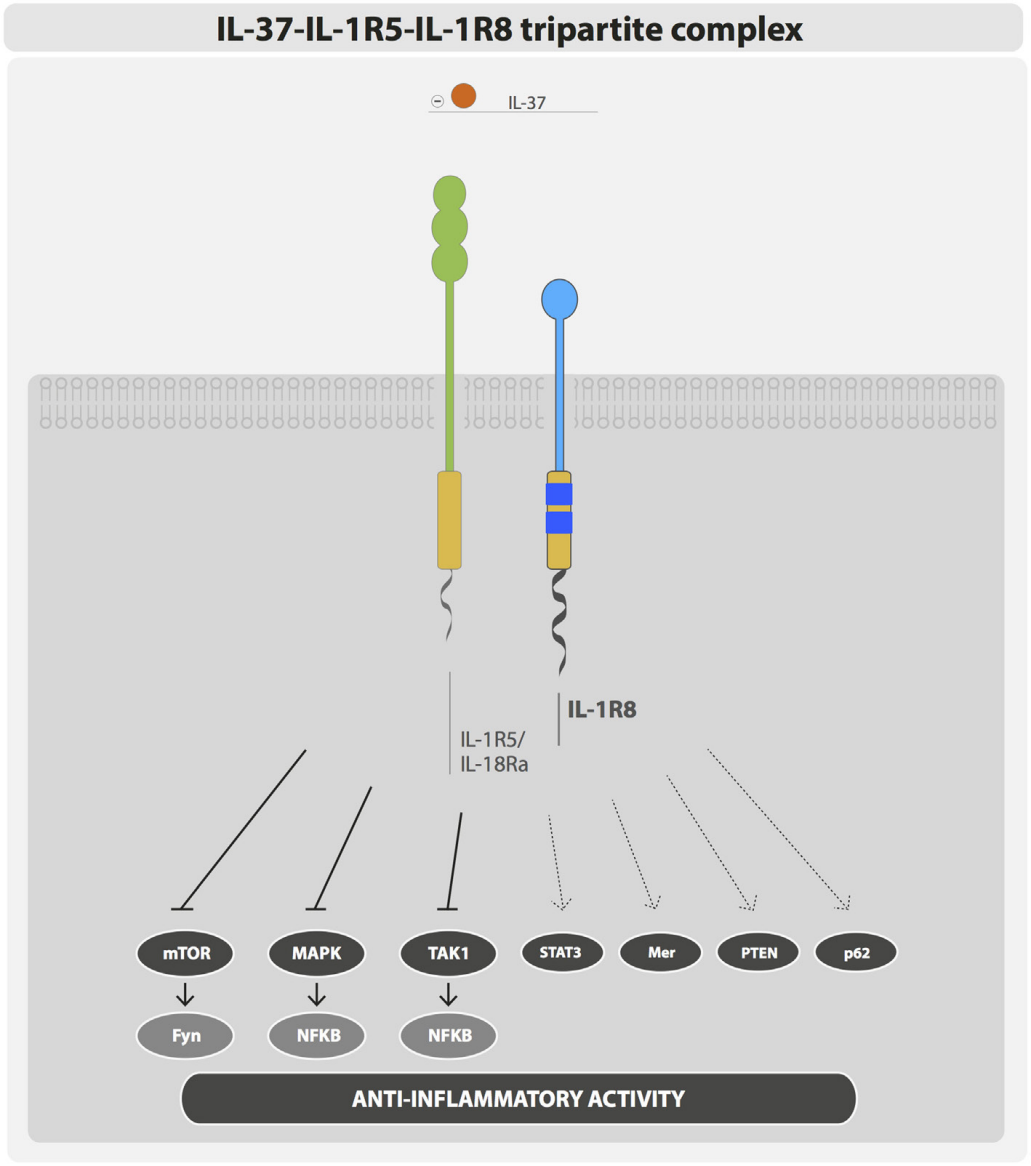
**IL-37–IL-1R5–IL-1R8 tripartite complex**. IL-37 anti-inflammatory activity is exerted through the formation of a membrane bound tripartite complex composed of IL-37, IL-1R5, and IL-1R8.

IL-1R8 was considered an orphan receptor, lacking a specific ligand. IL-37 has been recently demonstrated to bind IL-1R8 and to generate the tripartite complex IL-37–IL-1R5/IL-18Rα–IL-1R8 (11) (Figure 2). IL-37 is an anti-inflammatory cytokine that dampens the inflammatory response triggered by TLRs and cytokines in peripheral blood mononuclear cells (PBMCs), in macrophages and epithelial cells, and IL-37-transgenic mice (IL-37tg mice) are protected in different inflammatory pathological conditions (50). Recently, advanced imaging analysis revealed a rapid interaction of IL-37 with both IL-1R5/IL-18Rα and IL-1R8 in human PBMCs and bone marrow-derived macrophages (BMDMs) of IL-37tg mice upon stimulation with LPS (11). In particular, following a pro-inflammatory stimulus, the tripartite complex IL-37–IL-1R5/IL-18Rα–IL-1R8 was formed on the cell membrane of PBMCs and cell lines (RAW, HEK293, and A549). IL-1R8 and IL-1R5/IL-18Rα were both required to support the anti-inflammatory activity of IL-37 in PBMCs, THP-1 macrophages, and A549 epithelial cells. Indeed, silencing of IL-1R8 or IL-1R5/IL-18Rα or both in these cell types impaired the IL-37-mediated reduction of inflammatory cytokines (e.g., IL-β and TNF), upon stimulation with LPS. Finally, proteomic and transcriptomic analysis demonstrated that the IL-37–IL-1R5/IL-18Rα–IL-1R8 complex triggered multiple signaling events leading to anti-inflammatory responses, such as inhibition of MAPK, NFκB, mTOR, TAK1, and Fyn and activation of STAT3, Mer, PTEN, and p62(dok). Thus, IL-1R8 acts as a coreceptor for IL-1R5/IL-18Rα upon IL-37 binding, and it is required for the anti-inflammatory activity of IL-37 (11, 51) (Figure 2). This mechanism is relevant *in vivo*, since IL-1R8 deficiency abolished the protection of IL-37tg mice against endotoxin challenge or the protective role of IL-37 in a model of non-resolving *Aspergillus fumigatus* infection and pulmonary damage (11, 52). Moreover, in a model of OVA-induced asthma, IL-37-driven anti-inflammatory effects were abolished in mice lacking either IL-1R5/IL-18Rα or IL-1R8 (53).

IL-37-overexpressing mice showed improved response to insulin and increased glucose tolerance and were protected from obesity. In addition, in adipocytes and macrophages IL-37 activated AMPK (54). Since IL-37–IL-1R8 signaling inhibited mTOR signaling, whereas AMPK, STAT6, and transcription factors of the Foxo family were activated (11), these results indicate that the IL-37–IL-1R8 axis is also involved in regulating inflammation-dependent modification of cell metabolism, favoring a pseudo-starvational state in macrophages and DCs.

Thus, these results demonstrate that IL-1R8 is part of the receptor complex of the anti-inflammatory cytokine IL-37 and mediates an anti-inflammatory signaling activation.

## Regulation of ILR and TLR Signaling by IL-1R8

**Figure 3:**
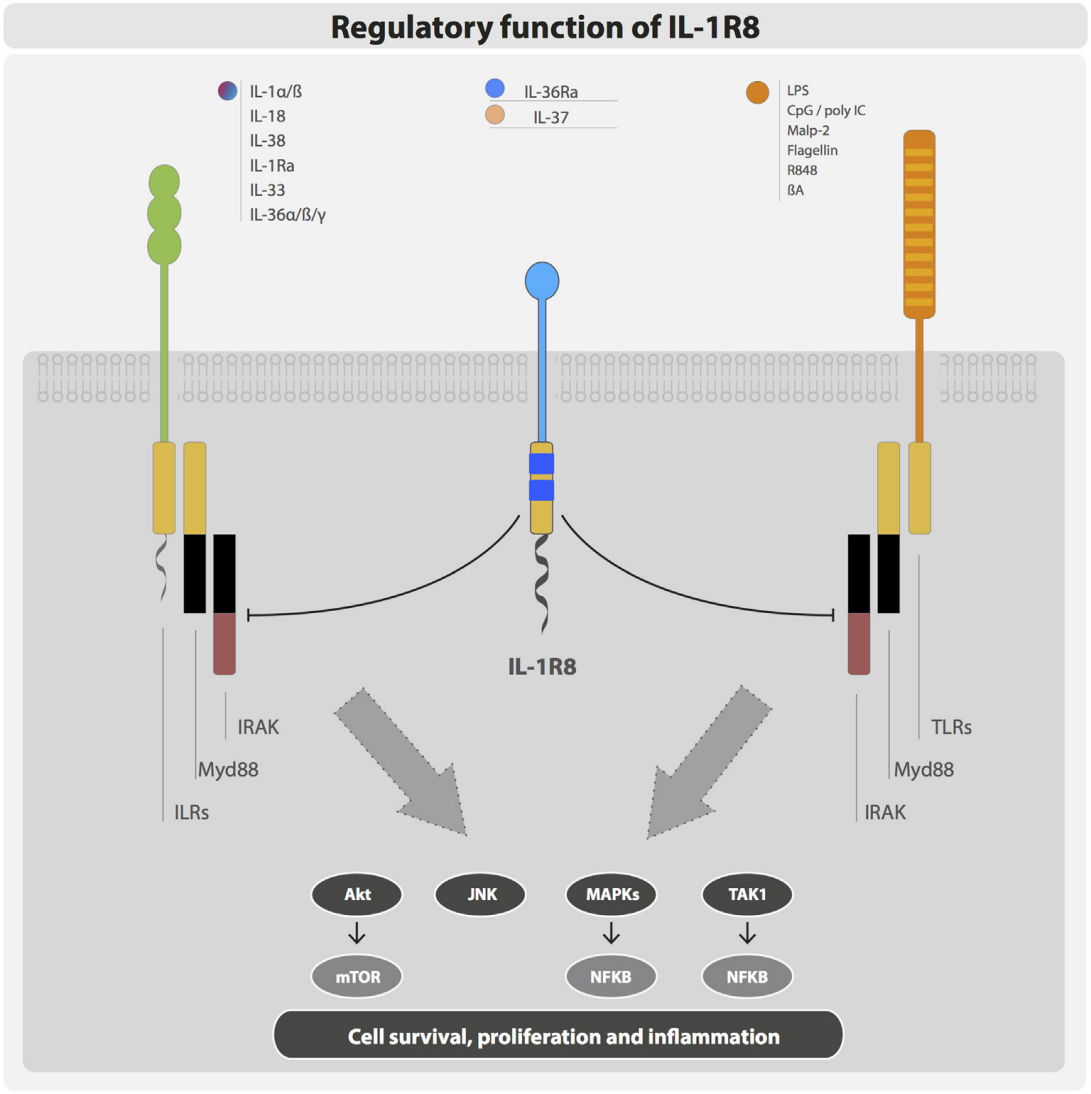
**Negative regulation exerted by IL-1R8**. IL-1R8 is composed of a single extracellular domain, a transmembrane domain, a cytoplasmic TIR domain, and an unusually long tail (95 residues). The IL-1R8 TIR domain lacks two conserved residues (Ser447 and Tyr536), which are replaced by Cys222 and Leu305 suggesting unconventional signaling. IL-1R8 acts as a negative regulator of ILR and TLR signaling, inhibiting TIR-containing receptors and adaptor proteins, and thus blocking Akt, JNK, MAPKs, TAK1, and consequently mTOR and NFκB activation.

IL-1R8 exerts its regulatory activity by inhibiting NFκB and JNK activation induced by TIR-containing ILRs or TLRs upon ligand binding, but not by other receptors such as TNF receptors. In particular, IL-1R8 was shown to tune the activation of IL-1R1, IL-1R5/IL-18Rα, IL-1R4/ST2, TLR4, TLR7, TLR9, TLR3, and TLR1/2 (29, 45, 55–57) (Figure 3).

The knowledge about the mechanism of inhibition exerted by IL-1R8 is still fragmentary. Upon stimulation with IL-1, the IL-1R8 extracellular domain was shown to block the dimerization between IL-1R1 and IL-1R3/IL-1RAcP, and the intracellular TIR domain was shown to bind the TIR-containing adaptor protein MyD88 and downstream signaling molecules (IRAK and TRAF6), thus modulating IL-1 signaling (55, 57). Similarly, the targeting of IL-1R4/ST2 was shown to be dependent on both the extracellular immunoglobulin and TIR domains (45). In contrast, only the TIR domain was necessary for the inhibition of TLR4 signaling, as demonstrated by mutagenesis studies (55, 57). Indeed, a nonsense mutation (Q111*) and a frameshift mutation (P2fs) cause the generation of a truncated form of IL-1R8, which lose inhibitory activity (55, 57, 58). A computational approach revealed that IL-1R8 targeting of TLR4 and TLR7 signaling was dependent on IL-1R8 intracellular TIR domain and in particular on the BB-loop, which is shared by all TIR domain-containing proteins. The model proposed showed that IL-1R8 binds through its BB-loop region to TLR4 and TLR7 interfering with the dimerization site and replaces a MyD88 monomer, thus disturbing MyD88 homodimerization (59). Recent computational studies that predict the three-dimensional structures of the TLR family proteins suggested that IL-1R8 does not block the formation of the Myd88-dependent signalosome, but it inhibits NFκB activation by preventing the translocation of the signalosome from the receptor (58). This strategy would be similar to that used by IRAK-M, which blocks the dissociation of the myddosome from the receptor (19).

Moreover, it was demonstrated that IL-1R8 could potentially interact with all TIR domain-containing proteins in the TLR pathway, preventing the dimerization of Mal, TRAM, and TRIF, and inhibiting signalosome formation. However, the BB-loop of IL-1R8 is not involved in the interaction with Mal and TRAM. Thus, in addition to MyD88-dependent pathway, IL-1R8 can inhibit TRIF-dependent signaling. Indeed, IL-1R8 was shown to target TRIF-dependent TLR3 signaling, probably by blocking TRAM homodimerization and TLR4–TRAM and TRIF–TRAM interactions (10, 58, 60). One of the clinically observed oncogenic mutations of IL-1R8 (L282M) is located on the IL-1R8–Mal and IL-1R8–TRIF interaction sites and abolishes the interaction with TRIF. These results indicate that the BB-loop of IL-1R8 is relevant in the interaction with TLRs, but not necessarily with other TIR-containing molecules (58).

c-Jun N-terminal kinase and mTOR phosphorylation were enhanced in IL-1R8-deficient Th17 cells. IL-1R8 is therefore crucial in the modulation of metabolism, differentiation, expansion, and effector functions of Th17 cells (61). IL-1R8 was also demonstrated to target mTOR phosphorylation driven by IL-1 or TLR agonists derived from commensal flora in intestinal epithelial cells (IECs) (62). IL-1R8 therefore emerges as a regulator of the cell cycle, playing a crucial role in homeostatic conditions (Figure 3).

The interaction between IL-1R8 and other receptors of the family is still poorly defined and sometimes contradictory. This could be due to posttranscriptional modifications in different cell types that can affect its functions.

Thus, these results indicate that IL-1R8 interferes with the formation of TIR domain signalosome, prevents the dimerization of receptors, AcP, and adaptor molecules, and blocks signal transduction.

## Role of IL-1R8 in Infection-Driven Inflammation

**Figure 4:**
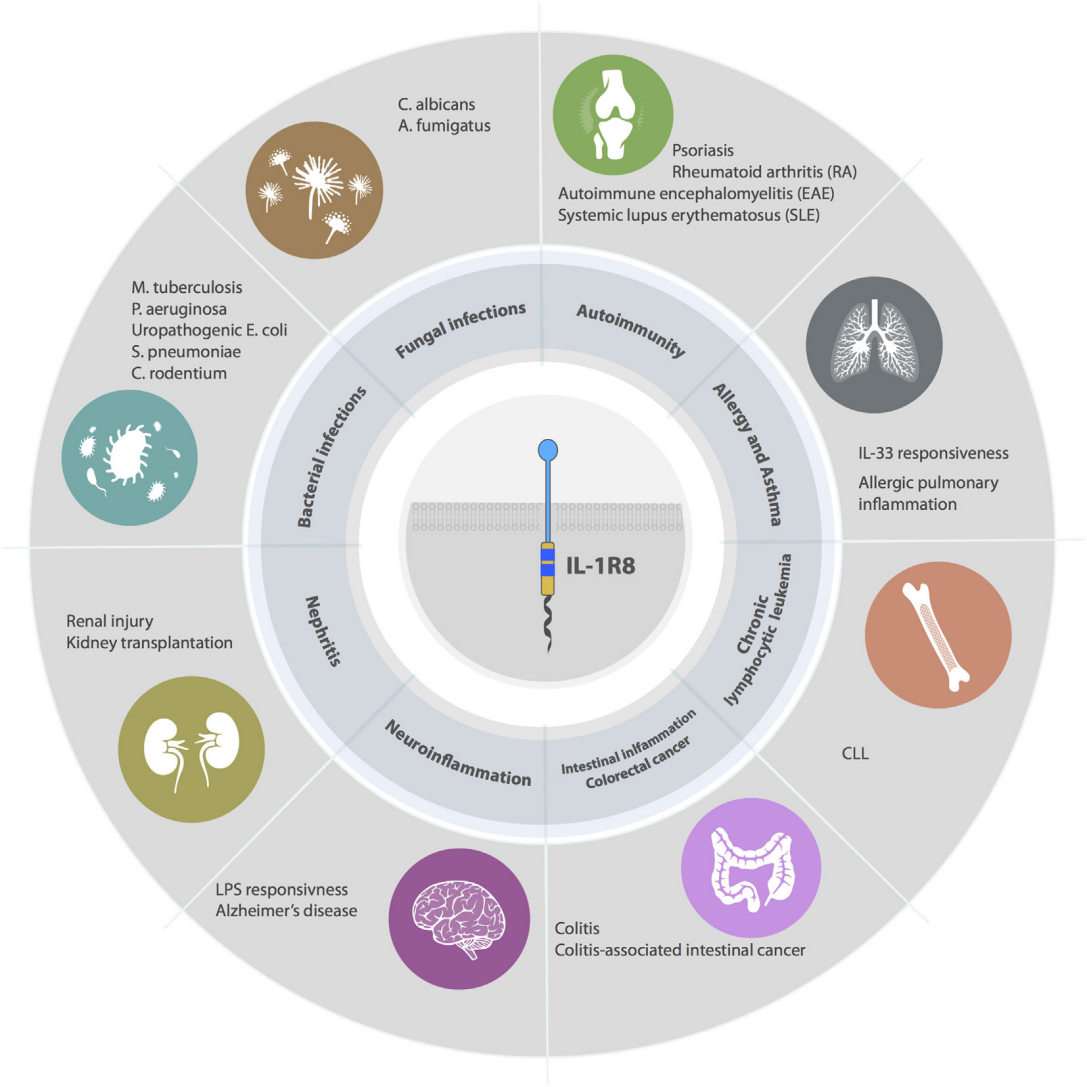
**Roles of IL-1R8 in pathology**. IL-1R8-deficient mice have demonstrated that IL-1R8 acts a key modulator of acute and chronic inflammation in several pathological contexts. For instance, IL-1R8 plays a non-redundant role in models of bacterial infections, fungal infections, autoimmune diseases, allergy, asthma, renal inflammation, brain inflammation, intestinal inflammation, and cancer (colorectal cancer and CLL).

IL-1R8 emerged as a non-redundant molecule in infectious conditions, playing a key role in the regulation of TLR and ILR responses to pathogens by dampening inflammation and tissue damage (Figure 4).

In *Mycobacterium tuberculosis* infection, IL-1R8-deficient mice displayed higher mortality compared with the control group, even if no difference in tissue bacterial load in the lung, liver, or spleen was observed. The increased susceptibility was dependent on the exacerbated systemic inflammatory response. Indeed, IL-R8-deficient mice presented an overwhelming inflammatory response, which is characterized by enhanced macrophage and neutrophil lung infiltration and increased systemic levels of inflammatory cytokines. The *in vivo* depletion of crucial inflammatory mediators (IL-1 and TNFα) in *M. tuberculosis* infection significantly prolonged the survival of IL-1R8-deficient mice (63). In a model of keratitis induced by *P. aeruginosa*, IL-1R8 was involved in the regulation of IL-1R1 and TLR4 signaling in T cells and dampening Th1 response, thus preventing tissue damage and promoting resistance to infection (64). Similarly, in acute lung infections caused by *P. aeruginosa*, IL-1R8-deficient mice showed increased susceptibility to the pathogen, in terms of mortality and bacterial load, and increased production of pro-inflammatory cytokines, both locally and systemically. The enhanced susceptibility was dependent on the deregulation of IL-1 signaling, since IL-1R1 deficiency abolished the phenotype observed in IL-1R8-deficient mice. IL-1R8 therefore emerged as a non-redundant regulator of IL-1 mediated control of *P. aeruginosa* infection (39).

In *C. albicans* and *A. fumigatus* infections, the absence of IL-1R8 led to increased susceptibility to mucosal and disseminated or lung infections (65). IL-1R8-deficient mice showed increased mortality and fungal burden, enhanced activation of IL-1 signaling and Th17 cell response, and reduced Treg activation. This correlated with higher levels of IL-12, IL-23, IL-6, IFN-γ, and IL-17 and reduced levels of IL-10. IL-1R8 was demonstrated to be a regulator of Th17 cells and IL-17A production by γδ T cells and a modulator of T cell polarization. The phenotype observed in IL-1R8-deficient mice could be due to a deregulated Th17 response, which is dependent on the uncontrolled IL-1 signaling. Indeed, *in vitro* experiments demonstrated that IL-17 production by CD4^+^ T cells, primed with *Candida*-pulsed DCs, was reduced by the treatment with IL-1β- and IL-23-blocking antibodies (65).

The role of IL-1R8 is therefore attributable to the negative regulation of IL-1 signaling in *P. aeruginosa*, *C. albicans*, and *M. tuberculosis* infections, since IL-1 neutralization was sufficient to abolish the phenotype in IL-1R8-deficient mice.

Moreover, IL-1R8-deficient mice on a BALB/c background showed enhanced mortality, upon endotoxin challenge (55). In line with this, IL-1R8 overexpression in lung epithelial cells reduced the inflammatory response and improved the survival of BALB/c mice in a model of LPS-dependent acute lung injury (66). Since IL-1R8-deficient mice on a C57BL/6 × 129/Sv background revealed no difference in terms of LPS reaction, compared with controls, it has been hypothesized that IL-1R8-mediated regulation of LPS response may depend on the background of mice (56).

The relevance of these data in the mouse was supported by a case-population study design in Vietnam, showing that 3 SNPs (rs10902158, rs7105848, rs7111432) in IL-1R8 gene correlated with the development of both pulmonary tuberculosis and tuberculous meningitis. Moreover, coinheritance of these SNPs with previously identified polymorphisms in TLR2 and TIRAP was associated with enhanced risk of susceptibility (67).

The protective role of IL-1R8 in the infections mentioned above is due to the regulation of ILR and TLR signaling that potentially cause detrimental inflammation and tissue damage. However, in a model of experimental urinary tract infection (UTI) induced by uropathogenic *E. coli*, IL-1R8-deficient mice displayed reduced renal bacteria outgrowth and diminished renal dysfunction UTI. IL-1R8 also modulated the recruitment of immune cells in the kidney, since a more sustained renal neutrophil influx was observed in the early phase of infection in IL-1R8-deficient mice. This is possibly due to the activity of IL-1R8 in dampening *E. coli* induced activation of tubular epithelial cells. Indeed, *in vitro* stimulation of IL-1R8-deficient tubular epithelial cells with LPS or heat-killed *E. coli* resulted in increased production of TNFα and chemokines (CXCL1, CCL2, and CCL3) and a mild increased expression of the adhesion molecule ICAM-1. Finally, IL-1R8 mRNA transcript was reduced in kidneys during the early phase of *E. coli* induced pyelonephritis (41). A recent *in vitro* study demonstrated that IL-1R8 regulates the responsiveness to LPS in a human BEC line, and, in line with previous studies, IL-1R8 mRNA and protein expression were downregulated upon stimulation with LPS (42). IL-1R8 silencing in BECs caused increased LPS-induced IL-6 and IL-8 production and this correlated with enhanced phosphorylation rate of JNK, p38, and ERK1/2. Finally, IL-1R8 siRNA transfected cells developed an impaired LPS tolerance, suggesting that IL-1R8 is involved in this process. Overall, these findings highlighted the importance of IL-1R8 in the negative regulation of urinary tract and renal response to bacterial infections (42).

Similar to what was observed in UTI, during pneumonia and sepsis induced by *Streptococcus pneumoniae* in the mouse, IL-1R8 deficiency was associated with delayed mortality, reduced bacterial load in the lungs, and reduced dissemination of the infection (68). Increased interstitial and perivascular inflammation was observed in IL-1R8-deficient lungs mice in the early phase of infection. Thus, IL-1R8 suppressed the protective antibacterial immune response in *S. pneumoniae* induced pneumonia (68).

Murine models of *Citrobacter rodentium* infection resemble human intestinal infections driven by enteric bacterial pathogens, such as enterohemorrhagic *E. coli* (EHEC) and *Salmonella typhimurium*. Upon *C. rodentium* infection, IL-1R8 deficiency correlated with accelerated IEC proliferation and enhanced pro-inflammatory and antimicrobial response. However, IL-1R8 was shown to be protective in this model, in terms of weight loss, intestinal damage, colitis score, and intestinal bacterial burden. IL-1R8 protective function was dependent on IL-1R1–MyD88 signaling, but not on TLR2 or TLR4 pathways, indicating that IL-1R1 may be the key receptor targeted by IL-1R8 in this context. Infected IL-1R8-deficient mice underwent a more rapid and dramatic loss of commensal flora, compared with controls. In infected mice, the microbiota depletion was directly dependent on the exacerbated antimicrobial response occurring in IL-1R8-deficient mice that favored pathogen colonization. Thus, IL-1R8-mediated regulation of IECs is responsible for the inhibition of a strong antimicrobial response that would otherwise lead to a rapid depletion of the commensal microbiota, during intestinal infection. In turn, the absence of competing microflora would favor the colonization by bacterial pathogens (69).

These studies indicate that IL-1R8 plays a crucial role in favoring the maintenance of a delicate equilibrium between the protective immune response against infections and the development of detrimental inflammation and host injury. The activity of IL-1R8 is therefore strictly dependent on the context and several lines of evidence suggest that during homeostasis the constitutive expression of IL-1R8 protects against inappropriate responses, whereas its downregulation during acute inflammatory stimulation enhances the effectiveness (and pathogenic potential) of antibacterial host defense.

## Role of IL-1R8 in Autoimmunity and Allergy

The interest in studying IL-1R8 involvement in autoimmunity arises from the fact that ILRs and TLRs are key players in the pathogenic mechanisms of autoimmune disorders (Figure 4). In particular, IL-1 regulates the differentiation and function of Th17 cells, which are involved in inflammatory diseases such as rheumatoid arthritis (RA), multiple sclerosis, psoriasis, and inflammatory bowel disease (IBD) (70). IL-33 is a driver of type 2 inflammatory responses and is implicated in allergy and asthma (71).

Gulen et al. recently showed that IL-1R8 was induced during Th17 cell polarization and that it controlled Th17 cell differentiation, expansion, and effector functions through the direct inhibition of IL-1 signaling in T cells. An increased phosphorylation rate of JNK, mTOR, and 4EBP1 was observed in IL-1R8-deficient T cells, upon stimulation with IL-1. In particular, IL-1-induced mTOR pathway was critical for the IL-1R8-mediated modulation of Th17 response. Thus, IL-1R8 emerged as a key regulator of IL-1 activity in Th17 cells, and it was also observed to be involved in the Th17-mediated development of central nervous system (CNS) autoimmune disorders. Indeed, IL-1R8-deficient mice revealed higher susceptibility to experimental autoimmune encephalomyelitis (EAE), due to an increased Th17 infiltrate in the CNS and enhanced Th17 polarization and pathogenic functions (61).

Toll-like receptors and ILRs are involved in the pathogenesis of RA (72–74). IL-1R8 was shown to suppress the spontaneous release of cytokines in human RA synovial cells *in vitro*, suggesting its involvement in the modulation of chronic inflammation in RA. *In vivo* experiments supported this evidence, since IL-1R8-deficient mice developed a more severe disease in both zymosan-induced arthritis and collagen antibody-induced arthritis models, which was associated with increased cellular infiltration into the affected joints. IL-1Ra treatment reduced the susceptibility of IL-1R8-deficient mice in zymosan-induced arthritis, suggesting that IL-1 played a central role in this model. However, the phenotype of IL-1R8-deficient mice was not completely rescued by the treatment, possibly because other TLR (e.g., TLR2) or ILR ligands are implicated in zymosan-induced arthritis pathogenesis (74). In agreement with this study, IL-1R8 expression was reduced in peripheral blood of patients with psoriatic arthritis, compared with healthy donors (36). Moreover, IL-1R8 deficiency caused enhanced susceptibility to psoriasis, associated with increased infiltration and activation of γδ T cells, in both Aldara- and rIL-23-induced psoriasis models. Interestingly, IL-R8 directly modulated IL-1-driven IL-17A expression by γδ T cells *in vitro* and *in vivo*, and IL-17A depletion abolished the phenotype observed in IL-1R8-deficient mice (75).

Increasing evidences implicated IL-1R8 in the pathogenesis of systemic lupus erythematosus. Indeed, altered TLR signaling in DCs and B cells is one of the driving mechanisms of this autoimmune disorder. In particular, immune complexes containing the lupus autoantigen U1snRNP or nucleosomes activate DCs and autoreactive B cells *via* TLR7 and TLR9, respectively (76, 77). In the mouse, IL-1R8 deficiency alone did not induce autoimmunity against DNA. However, IL-1R8 deficiency in C57BL/6^lpr/lpr^ mice, which develop delayed autoimmunity due to impaired Fas-induced apoptosis of autoreactive B and T cells, caused increased activation of DCs and B cells and production of pro-inflammatory cytokines (CCL2, IL-6, and IL-12p40) and B cell antiapoptotic mediators (Baff/BlyS and Bcl-2). Moreover, IL-1R8 regulated B cell proliferation, upon exposure to RNA and DNA immune complexes or other TLR agonists. IL-1R8-deficient C57BL/6^lpr/lpr^ mice also displayed and increased production of autoantibodies (anti-dsDNAIgG, anti-nucleosome, anti-Sm antigen, anti-snRNP, and rheumatoid factor) and presented a massive lymphoproliferative disorder, associated with enhanced autoimmune lung disease, lupus nephritis, and hypergammaglobulinemia, compared with IL-1R8-competent C57BL/6^lpr/lpr^ controls (78). In line with this, IL-1R8 was also protective in a model of hydrocarbon oil-induced lupus, in which it modulated TLR7-mediated activation of DCs and expansion of autoreactive lymphocyte clones. IL-1R8 is therefore involved in the regulation of DC and B cell activation, by preventing exacerbated autoimmune reactions, lymphoproliferation, and tissue damage in SLE (79). The data in the mouse were supported by recent analysis of IL-1R8 involvement in SLE in human. A case study of a cohort of SLE patients revealed a reduced frequency of IL-1R8^+^ CD4^+^ T cells in the peripheral blood of SLE patients compared with healthy individuals. Moreover, the frequency of IL-1R8^+^ CD4^+^ T cells was further reduced in SLE patients with nephritis, compared with those without nephritis (80). Zhu et al. showed that B cells from SLE patients displayed an upregulation of TLR7 and TLR9 compared with healthy controls, but the response to corresponding ligands was normal or even reduced. The authors suggested that this could be explained by the enhanced IL-1R8 expression in SLE B cells, even though the pathological significance of IL-1R8 increase in this context is still unclear (81). A genetic analysis of allelic variants of the IL-1R8 gene in a large European-descent population showed no correlation between IL-1R8 polymorphisms and SLE, but the analysis was restricted to a single missense SNP (rs3210908) (82). More recently, another genetic variant of IL-1R8 (rs7396562) was identified, and it was demonstrated to correlate with the susceptibility to SLE, in a Chinese population (83).

IL-33 signaling is a key driver of type 2 immunity, which favors protective immune responses in parasite infections and tissue repair but is also involved in pathological conditions, such as asthma, allergy, and eosinophilia (84). IL-33 receptor (IL-1R4/ST2) affects innate and adaptive lymphoid cells (ILCs and Th2), inducing the production of type 2 cytokines (IL-4, IL-5, IL-13), and can be targeted by IL-1R8. Indeed, IL-1R8 inhibits IL-33-mediated signaling in Th2 cells, controlling the production of type 2 cytokines *in vitro* and *in vivo* (45). IL-1R8-deficient mice were shown to be hyper responsive to IL-33, in terms of lung inflammation, splenomegaly, and increased serum levels of IL-5 and IL-13 (45). Moreover, in a model of allergic pulmonary inflammation induced by OVA, IL-1R8 deficiency was associated with increased leukocyte lung infiltration, IL-5 and IL-4 levels, and OVA-specific IgE induction, due to an exacerbated Th2 response (45). These results indicate that IL-1R8 serves as a negative feedback control in Th2 polarization and restimulation, thus controlling allergic inflammatory responses. However, a genetic study performed on a cohort of Japanese asthma patients revealed that none of the alleles or haplotypes of IL-1R8 identified were associated with asthma susceptibility or asthma-related conditions (85).

These data demonstrate the relevance of the control mediated by IL-1R8 on T and B lymphocytes and on antigen-presenting cells in the development of autoimmune and allergic diseases.

## Role of IL-1R8 in Kidney Sterile Inflammation

IL-1R8 is expressed at high levels in the kidney, in particular, by tubular epithelial cells and immune cells such as DCs and macrophages. Immunohistochemical analysis revealed extensive IL-1R8 positivity in the majority of tubular epithelial cells of the renal cortex, showing a predominant expression at the apical side of renal proximal tubules (29). IL-1R8 was shown to be a key player in sterile kidney diseases, by regulating TLR activation by nucleosomes and DAMPs, released during ischemic cell necrosis and associated with pathological conditions, such as lupus nephritis, postischemic acute renal failure, or kidney transplantation (78, 79, 86, 87) (Figure 4).

In a postischemic renal failure model, IL-1R8 deficiency was associated with increased renal injury, due to a massive activation of myeloid cells, increased intrarenal cytokine and chemokine production and increased leukocyte recruitment. In this model of sterile inflammation, DAMPs activate immune cells, in particular, neutrophils and macrophages, mainly *via* TLR4 and TLR2. In a model of renal ischemia/reperfusion, bone marrow chimeric mice demonstrated a major role of IL-1R8 in the hematopoietic compartment, since Il1r8^+/+^ animals transplanted with Il1r8^−/−^ hematopoietic cells reproduced the phenotype of IL-1R8-deficient mice (86). In line with this, in a mouse model of fully mismatched kidney allotransplantation, IL-1R8-deficient grafts were less tolerated compared with control grafts, leading to acute rejection. Moreover, IL-1R8 deficiency was associated with an enhanced ILR- and TLR-driven posttransplant kidney inflammatory response, in particular, due to increased neutrophil and macrophage infiltrate and higher expression of TNFα and chemokines. An amplified adaptive response was also observed in IL-1R8-deficient mice, in which expansion and maturation of DCs was enhanced and the immune response against donor antigens was exacerbated. The higher allostimulatory activity of DCs may possibly explain the increased frequency of reactive T cells and reduced Treg development in absence of IL-1R8. Thus, IL-1R8 plays a key role in the regulation of the allogeneic immune response *in situ* and is involved in graft survival (87).

In case of renal fibrosis induced by unilateral ureteral obstruction (UUO), IL-1R8 deficiency did not modulate the renal pathology. Indeed, IL-1R8-deficient mice did not show any difference compared with controls in this model, in terms of mRNA transcript of pro-inflammatory and profibrotic mediators, leukocyte recruitment, and renal injury (88). These data are in line with the evidence that TLR2, TLR9, and MyD88 signaling are not involved in the pathogenesis of postobstructive renal fibrosis (89).

## Role of IL-1R8 in Brain Inflammation

IL-1R8 is expressed in the brain by neurons, microglia, and astrocytes, and it was shown to be involved in the regulation of LPS responsiveness in the brain (16, 33, 90) (Figure 4). Indeed, IL-1R8 deficiency was associated with a massive LPS-induced inflammation in the brain. In response to LPS, IL-1R8 negatively regulated CD40, ICAM, and cytokine (IL-6 and TNFα) mRNA expression in microglial cells and cytokine production in hippocampal tissue. This is in line with an increased hippocampal expression of CD14 and TLR4, and NFκB activation in IL-1R8-deficient mice (91).

In addition, it has been observed that cognitive and synaptic functions, such as novel object recognition, spatial reference memory, and long-term potentiation (LTP), were impaired in IL-1R8-deficient mice, in absence of any external stimulus. This was associated with a higher expression of IL-1R1 and TLR4 and an enhanced activation of IL-1R1 and TLR4 downstream signaling molecules (IRAK1, c-Jun, JNK, and NFκB) (92). Indeed, treatment with IL-1Ra and anti-TLR4 antibody and the inhibition of JNK and NFκB rescued the deficit in LTP in IL-1R8-deficient animals, suggesting a central role of IL-1R1 and TLR4 signaling in this model. IL-1α and high mobility group box 1 (HMGB1), which activate IL-1R1 and TLR4, respectively, were proposed to play a central role in the phenotype observed and the expression levels of both molecules were increased in IL-1R8-deficient mice. These findings revealed a key role of IL-1R8 in modulating the inflammatory response associated with synaptic and cognitive decline and identified IL-1α and HMGB-1 as central mediators in this process (92). Moreover, IL-1R6 antagonist (IL-36Ra) inhibits the IL-1- and LPS-induced inflammatory response in glial cells, and this effect was absent in mixed glia prepared from IL-1R8-deficient mice, suggesting the involvement of IL-1R8 in the anti-inflammatory activity of IL-36Ra, possibly mediated through the production of IL-4 (16). Finally, a recent study showed that IL-1R8 acted as a negative regulator of β-amyloid (Aβ) peptide-induced TLR2 signaling in the brain. Aβ is the main component of neuritic plaques in Alzheimer’s disease (AD) and the primary mediator of the AD-associated neuroinflammation (49). The response to the TLR2 agonist (Pam_3_Cys_4_) was increased in glial cells from IL-1R8-deficient mice and Aβ-induced inflammation in the brain was enhanced in IL-1R8-deficient mice, in terms of cytokine production (IL-6 and TNF-α). *In vitro* experiments demonstrated that Aβ treatment increased the expression of TLR2 and decreased the expression of IL-1R8 in microglia and this was mimicked by the treatment with the TLR2 agonist Pam_3_Cys_4_. Anti-TLR2 treatment of microglia attenuated the inflammatory response and the impairment in LTP, both induced by Aβ, confirming the central role of TLR2 in the Aβ-induced neuroinflammation. Interestingly, TLR2 neutralization also led to an increase of IL-1R8 mRNA (49). These findings highlighted the key role of IL-1R8 in the modulation of TLR2-induced inflammation in the brain and its relevance in a potential therapeutic approach targeting TLR2 in AD-related pathology.

## Role of IL-1R8 in Intestinal Inflammation and Intestinal Cancer

IL-1R8 was demonstrated to be a key regulator of intestinal homeostasis (Figure 4). IECs are intrinsically hyporesponsive to bacterial products, thus not only preventing exaggerate inflammatory responses against the commensal flora but also limiting the enteric host defense (56). On the other hand, gut microflora-mediated activation of ILRs and TLRs provides the survival signals for IECs and this pathway is targeted by IL-1R8, which is therefore involved in controlling proliferation and survival in colon crypts (93). IL-1R8-deficient mice displayed constitutive NFκB and JNK activation and increased expression of Cyclin D1 and Bcl-xL. The effect was further enhanced upon treatment with IL-1 or LPS, and it was dependent on the commensal flora, since microbiota depletion rescued the phenotype (93). This phenotype in healthy mice was not confirmed by other studies (56, 94), probably because of the animal house-dependent variation of the microflora. The relevance of IL-1R8 expression in IEC in terms of response to intestinal infections and control of commensal microbiota has been described above.

In dextran sodium sulfate (DSS)-induced colitis, IL-1R8 deficiency is associated with an exacerbated intestinal inflammation, in terms of weight loss, intestinal bleeding, local tissue damage, and a reduced survival. This correlates with an increased leukocyte infiltration in the intestine and higher level of pro-inflammatory cytokines (TNFα, IL-6, IL-1β, IL-12p40, IL-17), chemokines (CXCL1, CCL2), and prostaglandins. Experiments with bone marrow chimeric mice demonstrated that the regulatory function exerted by IL-1R8 occurs in epithelial cells, in both DSS- and enteric pathogen-induced colitis (56, 93).

Epidemiological studies have shown that chronic inflammation, both dependent on infectious agents or not, can increase the risk of cancer. The hallmarks of cancer-related inflammation are comparable to those observed in chronic inflammatory conditions: inflammatory cells and mediators are present in the tumor tissue, and they are implicated in tissue repair, remodeling, and angiogenesis. This “smoldering inflammation” occurs even in tumors that are not directly caused by an inflammatory trigger. Cancer-related inflammation depends on two possible pathways: an intrinsic pathway, driven by oncogenic mutations that cause both neoplasia and inflammation, or an extrinsic pathway, driven by inflammatory conditions that favor tumor development (e.g., colitis-associated intestinal cancer) (95–98). Several studies have revealed a crucial role of ILR and TLR signaling in this context, in which NFκB is one of the key orchestrators, and that IL-1R8 plays a protective role in the pathogenesis of cancer-related inflammation in different murine models of colon cancer (98). IL-1R8 was studied in a model of CAC, induced by the treatment with the procarcinogen azoxymethane (AOM), followed by DSS, which favors chronic inflammation (93, 94). This model mimics intestinal cancer that develops in chronic IBD patients, in particular, ulcerative colitis patients. In the AOM–DSS CAC model, IL-1R8 deficiency was associated with exacerbated inflammation in the intestine, leading to increased susceptibility to cancer development, in terms of number, size, and severity of lesions. IL-1R8 negatively regulated intestinal permeability, *in situ* production of pro-inflammatory cytokines and chemokines and prostaglandin E_2_, and the expression of NFκB-induced genes involved in cell survival and proliferation (Bcl-xL and Cyclin D1) (93, 94). In this context, chemokines favored cancer progression, influencing the extent and type of leukocyte infiltrate (e.g., recruiting Th2 and Treg cells) and driving tumor cell and endothelial cell growth and migration (99, 100). Moreover, increased levels of IL-10 and TGF-β were observed in tumors of IL-1R8-deficient mice, reflecting an immunosuppressive microenvironment that inhibited T cell-dependent antitumoral immunity (100). The expression of IL-6, which promotes cancer growth, was also increased in the intestine of IL-1R8-deficient mice (101, 102). IL-1R8 overexpression in gut epithelial cells abolished the susceptibility of IL-1R8-deficient mice to CAC development, suggesting that the regulatory activity of IL-1R8 in IECs plays a central role in this model (93). Since commensal microflora-derived stimuli are necessary for the homeostasis of colon epithelium and are involved in colitis-associated carcinogenesis, IL-1R8 regulation may be possibly dependent on its direct modulation of microbiota-activated TLRs (103). However, IL-1R8-mediated targeting of other TLR- and ILR-related pathways cannot be excluded in this model.

IL-1R8 involvement in colon cancer was also investigated in the genetic Apc^min/+^ model, in which tumor initiation is caused by loss of heterozygosity (LOH) of the tumor suppressor Apc and which mimics the familial adenomatous polyposis syndrome (104). In Apc^min/+^ mice, IL-1R8 deficiency led to an increased susceptibility to cancer development, due to a more sustained activation of the Akt/mTOR pathway, which plays a crucial role in tumor initiation (105). In agreement with the CAC model, commensal bacterial played a pivotal role in colonic tumorigenesis, suggesting that IL-1R8 regulation might occur through the inhibition of TLR signaling, even if mTOR enhanced activation was also observed upon stimulation of epithelial cells with IL-1. Thus, IL-1R8 exerts an antitumoral activity by suppressing IL-1- and TLR-induced mTOR-mediated cell cycle progression and consequent genetic instability (62).

A recent study has investigated the role of IL-1R8 in human colorectal cancer, demonstrating that colon tumors express lower level of IL-1R8 compared with healthy tissues and that IL-1R8 is frequently inactivated in human colorectal cancer (32). Indeed, Zhao et al. identified a dominant-negative isoform of IL-1R8 (IL-1R8^ΔE8^) and RNA sequencing data demonstrated that the expression level of this isoform increased in human colon cancer, compared with healthy tissue. The IL-1R8^ΔE8^ isoform originated from a transcript that lacks the exon 8 of the gene and exhibited compromised integrity of the TIR domain, increased retention in the cytoplasm, and reduced N-linked glycosylation. The cytoplasmic retention caused a decrease in the cell surface expression of IL-1R8 and a consequent loss of its inhibitory activity. Moreover, IL-1R8^ΔE8^ isoform was shown to be able to interact with full-length IL-1R8, acting as an antagonist of IL-1R8 and thus suppressing its function. To investigate the mechanism responsible for IL-1R8^ΔE8^ isoform synthesis in tumor cells, sequence analysis were performed and predicted that exon 8 would be intrinsically a “weak” exon, with high probability of exclusion. Exon 8 also displayed a binding site for CTCF, a factor that favors the inclusion of weak exons, and since the binding can be reduced by methylation, hypermethylation was proposed to be the strategy followed by cancer cells that leads to IL-1R8^ΔE8^ isoform expression. Indeed, treatment with decitabine, a methyltransferase inhibitor, reduced IL-1R8^ΔE8^ isoform expression. To model the impact of IL-1R8^ΔE8^ isoform in colon carcinogenesis in the mouse, gut epithelium-specific IL-1R8 transgenic mice were generated, expressing a mutant form of IL-1R8 (IL-1R8^N85/101S^) that mimics IL-1R8^ΔE8^ isoform or wild-type IL-1R8 as a control. In both AOM and AOM–DSS models, the presence of wild-type IL-1R8 in IECs protected the mice from the development of colon cancer. On the contrary, mice expressing IL-1R8^N85/101S^ isoform had the same phenotype as IL-1R8-deficient mice, suggesting that complex glycan modifications and cell surface expression are necessary for IL-1R8 functional activity *in vivo* (32). Thus, IL-1R8 alternative splicing is an escape mechanism adopted by tumor cells to inactivate IL-1R8 through the generation of a dominant-negative isoform.

## Role of IL-1R8 in Chronic Lymphocytic Leukemia

Both genetic defects and microenvironment stimuli contribute to chronic lymphocytic leukemia (CLL) development and progression. Moreover, factors originating from the microenvironment are involved in the selection and expansion of the malignant clone (106, 107). Human malignant B cells expressed lower levels of IL-1R8 mRNA than normal B cells, and accordingly, in the well-established transgenic mouse model of CLL (TCL1), CD19^+^ B cells expressed lower levels of IL-1R8 mRNA transcript, compared with controls (107–110). IL-1R8 deficiency did not affect B cell compartment in healthy mice, whereas it correlated with an earlier and more severe appearance of monoclonal B cell expansion and a reduced mouse life span in TCL1 transgenic mice, mimicking the aggressive variant of human CLL (110). These findings revealed IL-1R8 inhibitory role in CLL initiation and progression, even though the molecular mechanism is still unclear (Figure 4). Endogenous TLR or ILR ligands are known to be involved in CLL and may be candidate targets of IL-1R8.

## Concluding Remarks

It is well established that IL-1R8 acts as negative regulator of ILR and TLR signaling, which are key pathways involved in inflammation and immunity. Early studies are consistent with the fact that IL-1R8 is a conserved and widely expressed molecule that plays a key role in the modulation of inflammation, tissue damage, and host defense against infections, autoimmunity, and cancer. Its mechanism of action is probably dependent on the interaction with TIR domain containing-signaling molecules, preventing the signalosome formation and activation. Although IL-1R8 was considered an orphan receptor, IL-36Ra has been proposed as brain-specific IL-1R8 ligand. Recently, IL-1R8 has been shown to act as a coreceptor for IL-37–IL-1R5/IL-18Ra and to be required for the anti-inflammatory activity of IL-37 (11, 51, 52). Indeed, IL-37 needs IL-1R8 to trigger a rapid anti-inflammatory program, revealing a novel role for IL-1R8. In addition to its regulatory activity, IL-1R8 therefore emerges as a coreceptor molecule, which able to boost IL-37-mediated signaling. Since IL-37 ameliorates insulin resistance and obesity-induced inflammation (54), it will be important to address whether IL-1R8 is involved in these contexts, preserving glucose tolerance and insulin sensitivity and reducing inflammation in the adipose tissue.

IL-1R8 regulates the metabolism, activation and polarization of several innate and adaptive immune cell types, as well as of non-hematopoietic cells. Thus, it plays a non-redundant role in the regulation of both pathogen-induced and sterile inflammation, managing the delicate equilibrium between host defense and detrimental inflammation.

The IL-1 system and TLR ligands affect all cells of the immune system, as well as epithelial, endothelial, and stromal cells. Since IL-1R8 is expressed by most cell types, but its functional characterization is still incomplete, future studies will be important to identify the peculiar role of IL-1R8 in the regulation of ILR- and TLR-dependent activation in specific cell types.

The involvement of IL-1R8 in human pathologies needs to be further investigated, since it could emerge as a potential target in several inflammatory contexts. Results showing that IL-1R8 inactivation is an escape mechanism adopted by cancer cells in human colon cancer are of particular importance, since they represent the first strong genetic evidence of the relevance of IL-1R8 in human disease. Further analysis of IL-1R8 polymorphisms and epigenetic regulations of IL-1R8 gene represent important future directions to gain a more precise view of IL-1R8 involvement in human diseases.

Given the well-established role of IL-1R8 as a key anti-inflammatory molecule in a broad spectrum of contexts, its targeting holds promise of innovative therapies in several pathological conditions.

## Author Contributions

CG and MM wrote the manuscript. IB and AM critically revised the manuscript.

## Conflict of Interest Statement

The authors declare that the research was conducted in the absence of any commercial or financial relationships that could be construed as a potential conflict of interest.
